# Bowel function in a prospective cohort of 1052 healthy term infants up to 4 months of age

**DOI:** 10.1007/s00431-024-05625-0

**Published:** 2024-05-31

**Authors:** Terhi Solasaari, Katri Korpela, Sohvi Lommi, Sanni Hyvönen, Susanna Gardemeister, Laura Merras-Salmio, Anne Salonen, Willem M. de Vos, Kaija-Leena Kolho

**Affiliations:** 1 Faculty of Medicine, University of Helsinki, Helsinki, Finland; 2 Pediatric Clinic, Social Services and Health Care Division, Helsinki, Finland; 3 Human Microbiome Research Program, Faculty of Medicine, University of Helsinki, Helsinki, Finland; 4 Folkhälsan Research Center, Helsinki, Finland; 5 Department of Pediatrics, Tampere University Hospital, Tampere, Finland; 6 Children’s Hospital, University of Helsinki and HUS, Helsinki, Finland; 7 Laboratory of Microbiology, Wageningen University, Wageningen, the Netherlands; 8 Faculty of Medicine and Health Technology, University of Tampere, Tampere, Finland

**Keywords:** Child, Crying, Feces, Stool

## Abstract

The purpose of this study is to describe the defecation pattern of healthy infants up to 17 weeks of age. We included 1052 healthy term infants from the prospective HELMi cohort (NCT03996304). Parents filled in recurring online questionnaires on feeding, gastrointestinal function, and crying weekly for the first 17 weeks of life. Defecation frequency was highest at the age of 3 weeks (a median of 4 times/day, interquartile range (IQR) 2.9–5). At each time point, the median defecation frequency of breastfed infants was higher than that of infants receiving formula (e.g., at week 17 a median of 2 times/day, IQR 0.9–3.6, and a median of 1.1, IQR 0.6–1.4, respectively). The dominant color of the stool was most often yellow or light brown. Nearly black stools were reported in the first week of life in 3.4%. Nearly half (47.4%) of the infants had green stool color dominating for at least 1 week, with comparable frequency among breastfed (47.7%) and formula-fed (45.2%) infants. Green stools were associated with a higher defecation frequency (linear mixed-effect model *p* < 0.0001). Occasional blood in stool was reported in 9.3% and recurrent blood in 5.2% of the infants with no difference in stool consistency. Hard stools were rare (≤ 1%).

*     Conclusion*: This study enlightens the spectrum of defecation patterns in healthy term infants during the first 17 weeks of life. A better understanding of bowel function helps healthcare professionals distinguish normal from abnormal when addressing defecation, the color of stools, and the type of feeding.

**Table:** 

**What is Known:** *• Breastfed infants have more frequent and more yellow-colored stools than formula-fed infants.* *• Stools with green color are often suggested by the parents or even by medical professionals to indicate disease or discomfort in early life.*	
**What is New:** *• Nearly half of the healthy term infants had green stool dominating for at least one week during the first 17 weeks and occasional blood was reported in almost 10% of the infants during this period.* *• Data on normal variation in bowel function and stool may serve primary health care professionals when educating the families and caretakers of infants.*

**Supplementary Information:**

The online version contains supplementary material available at 10.1007/s00431-024-05625-0.

## Introduction

Infantile colic, regurgitation, and constipation are among the most common reasons for parents to contact a healthcare professional in the first months after birth [1–3]. Accordingly, these entities are the most common functional gastrointestinal disorders (FGIDs) in infants [4, 5]. It has been estimated that the worldwide prevalence of regurgitation, colic, and constipation is 8–30%, 3–20%, and 9–15% in infants, respectively [4, 5]. Infants with gastrointestinal symptoms may not always fulfill the criteria for FGID. However, alterations in bowel function and crying habits are apt to cause concern for parents and caregivers [6].

Distinguishing normal from abnormal may be challenging in infancy. Information on bowel function and stool consistency during the first months of life is limited [7–10]. Previously, the defecation pattern of 600 healthy term infants was described up to the age of 3 months [11]. As the main finding, breastfed infants had more frequent, softer, and more yellow-colored stools than formula-fed infants. The study also concluded that green stools in formula-fed infants should be considered normal. Our observations of clinical practice are that stools with green color are nevertheless often suggested by the parents or even by medical professionals to be an indicator of disease or discomfort in early life.

We used the opportunity of a comprehensive birth cohort HELMi to study the defecation and crying patterns in term infants followed up on a weekly basis with electronic questionnaires [12]. Our aim was to gain a better understanding of normal bowel function and improve knowledge of the characteristics of associated crying in infants during their first 4 months of life.

## Methods

### Study design and participants

The study population was derived from the prospective HELMi (Health and Early Life Microbiota) cohort including infants born full-term without known congenital defects. Details of the cohort and study protocol were previously published [12]. In short, pregnant women with singleton gestation were recruited mainly in the capital region of Finland between February 2016 and March 2018, before the COVID-19 pandemic. Altogether, 1052 families completed the data for baseline and thereafter provided weekly data online on the child’s nutrition, bowel function, crying, and health and care practices for the first 17 weeks that we utilized here (Table 1 and eTable1). The study retention rate was 96.5% (*n* = 1015) at 3 months [12–15], and 99% attended the follow-up at national well-baby clinics including monitoring of weight gain, growth, and development. At 12 weeks (*n* = 1003), 86% (*n* = 863) of the infants were exclusively or almost exclusively breastfed (96–100% of the diet consisting of breast milk), 12% (*n* = 117) were partially formula-fed, and 2% (*n* = 21) were exclusively formula-fed. Less than 0.5% of all infants received other than cow milk–derived formula [12]. By the end of the first year, 2.7% were diagnosed with cow’s milk allergy and 6.5% with atopy [15, 16].

**Table 1:** Background data of the study cohort

Number of study infants	1052	
Sex, *n* (%)		
Girl	520 (49.4)	
Boy	532 (50.6)	
Gestational age (weeks), mean (SD)	40 (1.2)	
Birth weight (g), mean (SD)	3559 (437)	
Height at birth (cm), mean (SD)	51 (1.8)	
Delivery mode, *n* (%)		
Vaginal delivery	876 (83)	
Antibiotics (yes)	208 (20)	
Cesarean delivery	176 (17)	
Antibiotics (yes)	176 (100)	
Parity, *n* (%)		
Firstborn	505 (48)	
Parental age, mean (SD)		
Mother	32.8 (4.0)	
Father/co-partner	34.8 (5.2)	
Maternal smoking during the pregnancy, *n* (%)		
Regularly	2 (0.2)	
Occasionally	4 (0.4)	
Education, *n* (%)	Mother	Father/co-partner
University	927(88)	768 (73)
Vocational school/upper secondary	117 (11)	245 (23)
Secondary school	8 (1)	24 (2)
Not available		15 (1)

#### Ethics

The study was approved by the ethical committee of The Hospital District of Helsinki and Uusimaa 349 (263/13/03/03 2015) in accordance with the Declaration of Helsinki. One or both guardians provided written informed consent.

### Bowel function, consistency, and color of stool

Parents reported bowel function and stool color of the infants every week. The daily defecation frequency was calculated by dividing the sum of weekly stools by seven. Parents evaluated typical stool consistency by using the Bristol Stool Form Scale (BSFS) including seven categories. Low scores indicate firm stool and slow transit, while high scores indicate loose stool and fast transit [17]. Parents assessed defecation difficulty and abdominal pain using a 100 mm visual analog scale (VAS) with word anchors at each end [12, 13, 16]. The rightmost position of the scale reflected an uneasy defecation or more pain. The position on the scale was later translated into a number. The presence of abdominal pain was also evaluated with a separate yes/no question.

To report the dominant stool color each week, parents selected the most accurate alternative of six categories (yellow, green, gray, nearly black, light, and dark brown). The presence of blood among the stools was a separate yes/no question.

### Analyses related to the group of infants with green stools

To compare defecation patterns and crying between infants with green stool and their controls, we formed a case–control set-up. Based on questionnaires, we identified children with green stools for at least 3 consecutive weeks over the 17 weeks. Then, we selected age-, sex-, and delivery mode–matched controls with yellow-colored stool. This case–control cohort comprised 444 children (150 cases with repeated green-colored stool and 294 matched controls; eTable 2).

### Crying and feeding related to bowel function

To assess whether bowel function (defecation frequency and difficulty) was associated with excessive crying, we first identified week 5 to be the age of most abundant daily crying in this cohort. Secondly, we constituted groups defined by the amount of daily crying at the age of 5 weeks as follows: group 1 with a daily crying time of 0–2 h (*n* = 779), group 2 with a daily crying time from 3 to 4 h (*n* = 174), and group 3 with a daily crying time of 5 h or more (*n* = 27) (eTable 2). Moreover, to compare defecation frequency between mainly breastfed (*n* = 927) and formula-fed infants, we defined formula-fed as those whose main nutrition was formula at least 14 weeks out of the 17 weeks (formula-fed, *n* = 31). The rest (*n* = 94) were undefined.

### Statistical analyses

For statistical analyses, we used the GraphPad Prism version 9.0 for Windows (GraphPad Software, San Diego, CA, USA) and the SPSS software program version 29 (IBM Corp., Chicago, IL, USA). The data are presented with mean and standard deviation (SD), median and interquartile range (IQR), or numbers/proportion (%). For single-time point analyses, we used the independent samples *t*-test, the Mann–Whitney test, and the Kruskal–Wallis test as appropriate. Multivariate linear mixed-effects (lme) models were used to analyze the whole weekly time series data (function lme in R library nlme). In these models, we included age (including a second-order polynomial for potential non-linear associations), sex, main feeding type (breastfeeding, breastmilk from a bottle, or formula), and stool consistency and color that week (eTable 1). Missing data were rare and, according to our assumption, at random. No corrections were made for missing data. The level of statistical significance was set at *p* < 0.05.

## Results

We studied the bowel function in 1052 healthy term infants (Table 1) using weekly online questionnaires for the first 17 weeks. The compliance was excellent, and the proportion of missing data on all the responses was low throughout the study period (ranging from 0.9–11.8% per studied item). As an example, the number of reports on stool frequency and color varied from 935 (week 16) up to 1039 (frequency) and 1045 (color) per week.

### Defecation frequency, color, and consistency of stools

**Fig. 1:**
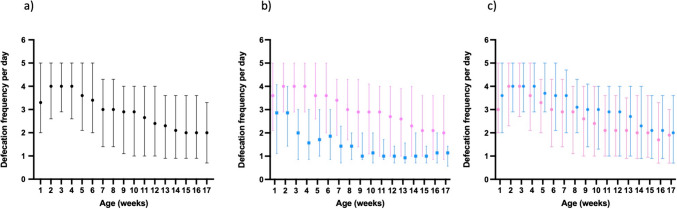
Defecation frequency in 1052 full-term infants for the first 17 weeks of life. The values represent the median and interquartile range. Defecation frequency in all infants (**a**), the effect of the feeding type (**b**), and sex (**c**) are presented. Breastfed infants (**b**) and girls (**c**) are marked in red (dots represent means) and formula-fed (**b**) and boys (**c**) in blue (squares represent means). The defecation frequency was reported weekly online throughout the study period

Defecation frequency was highest at the age of 3 weeks (a median of 4/day, IQR 2.9–5) followed by a steady decrease toward week 17 (a median of 2/day, IQR 0.7–3.3). The decline with age was highly significant (*p* < 0.0001 in lme). Defecation frequency was significantly higher among breastfed infants compared to formula-fed (lme *p* < 0.0001) (Fig. 1). Boys showed higher defecation frequency than girls (Fig. 1; lme *p* = 0.004).

**Fig. 2:**
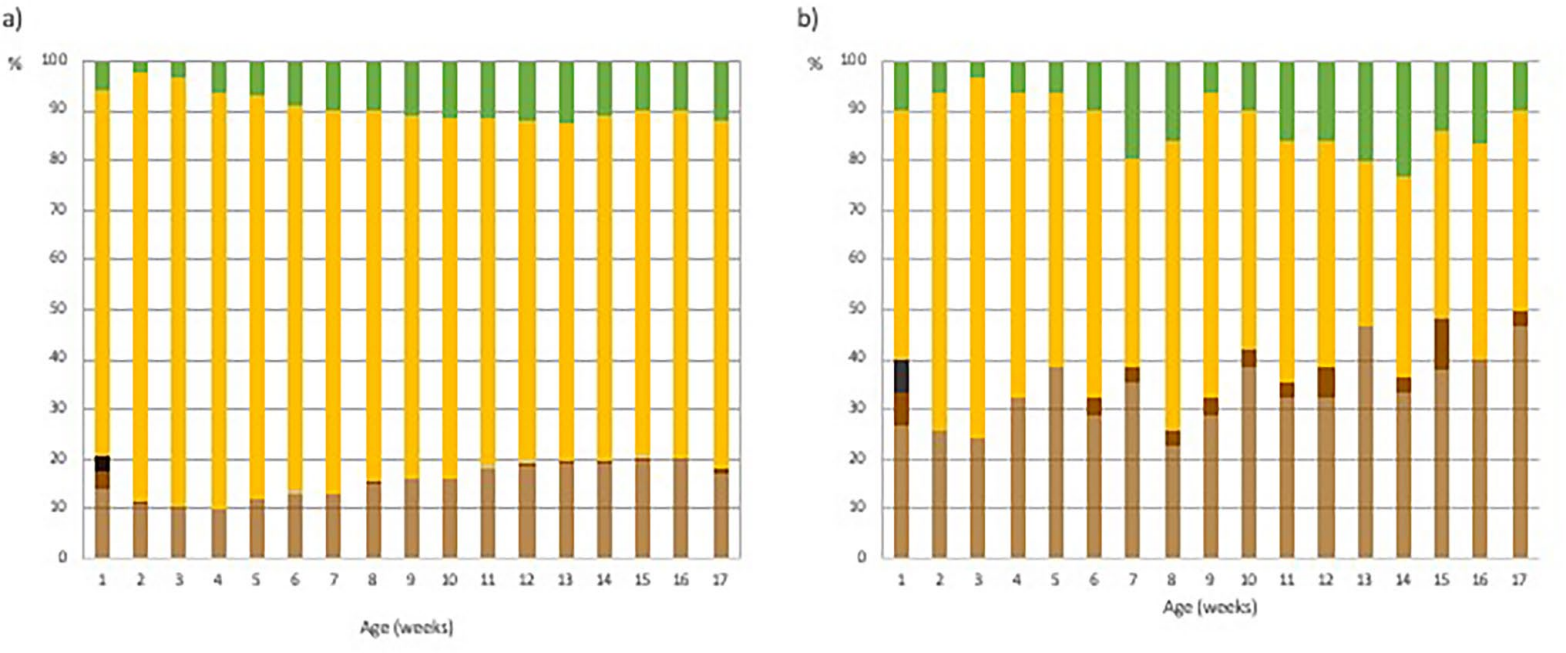
Stool color in full-term infants for the first 17 weeks of life. The data of the 1052 infants are shown separately for breastfed (**a**) and formula-fed (**b**) infants. The proportion of each color (light brown, dark brown, nearly black, gray, yellow, green) is presented in percentages. The predominant stool color was reported weekly online throughout the study period

Stools were most often yellow-colored (67–85% of infants) and light brown–colored (11–21% of infants). Stools in the breastfed group were more often yellow than in the formula-fed group (Fig. 2, *p* < 0.0001 *χ*^2^ test). Nearly black stools as the dominant stool color were reported in the first week of life in 3.4% of infants but rarely thereafter (≤ 0.1%). Brown or black stools were associated with significantly lower defecation frequency compared to yellow stools, while green stools were associated with a higher defecation frequency (lme *p* < 0.0001). Defecation frequency concerning diet and stool color are shown in eFigure1.

**Fig. 3:**
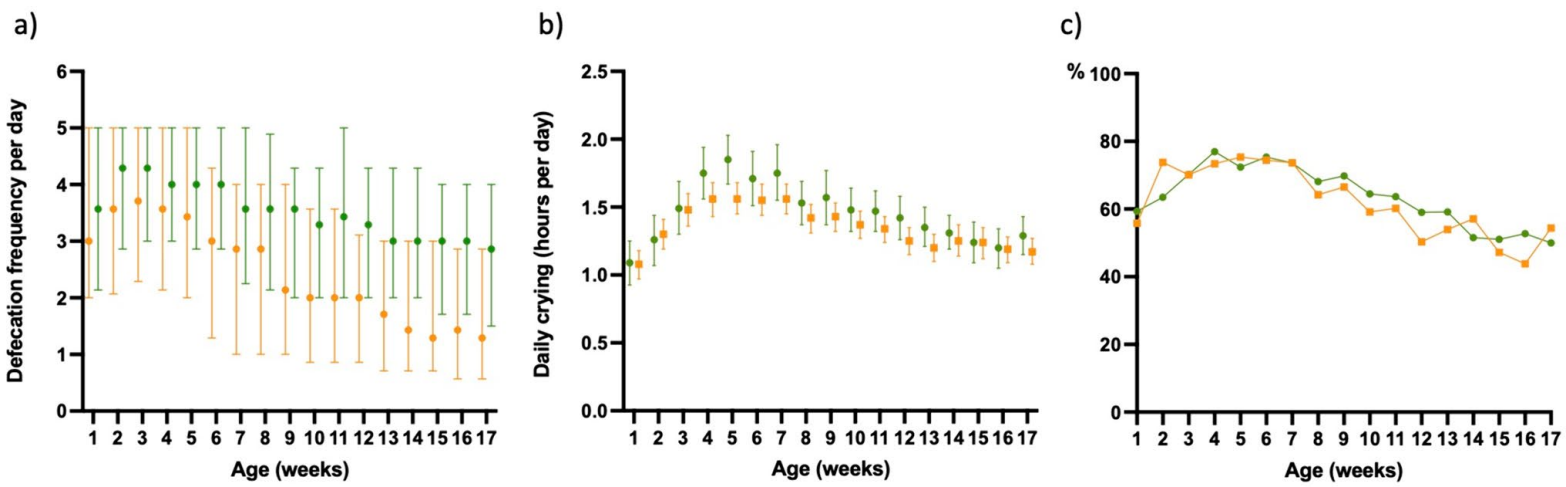
Defecation frequency, daily crying, and proportion of infants with abdominal pain related to stool color in full-term infants for the first 17 weeks of life. The group with green color (*n* = 150) presented predominately green stools during at least three consecutive weeks of life. The group with yellow stools (*n* = 294) presented with predominately yellow stools. Values in defecation frequency (**a**) represent the median and interquartile range, and values in daily crying (**b**) represent the mean and 95% confidence interval (marked with dots in the green group and squares in the yellow group). Abdominal pain (**c**) is presented as the proportion of infants each week reported to have pain

Nearly half (47.2%) of the infants had green as the dominant stool color for at least 1 week, with comparable frequency among breastfed (47.7%) and formula-fed (45.2%) infants. Single-week reports of green stools were seen in 17% (*n* = 179) of infants, while 30.4% (*n* = 320) reported green stool twice or more often. The median of observations was at week 11 with a significant non-linear association with age, initially increasing and then decreasing (lme *p* < 0.0001). Green stools were associated with looser stool consistency (lme *p* = 0.001) and higher defecation frequency (lme *p* < 0.0001). Infants with repeated green stools also showed higher defecation frequency (Fig. 3; statistically significant between weeks 4 and 17, *p*-values from < 0.0001 to 0.05) and more frequent abdominal pain than their controls, the latter difference being significant only in weeks 3 and 16 (*p*-values 0.006 and 0.015) (Fig. 3). However, in lme, the association between the intensity of abdominal pain and stool color was non-significant (*p* = 0.88). Reports of daily crying were comparable between green and yellow stool groups except in week 5 (more crying in the group with green stools, *p* = 0.0047, Fig. 3). When comparing weight at 3 months, there was no difference between these infants with green stool and yellow stool (mean weight 6.2 kg (SD 0.78) and 6.3 kg (SD 0.84) respectively, *p* = 0.171).

Stool consistency was mostly mushy (BSFS type 6) or runny (type 7), and the average consistency remained rather stable for 17 weeks (mean 6.0. (SD 0.47) with no difference between breastfed and formula-fed infants (data not shown)). Overall, hard stools (BSFS type 1) were rare in both feeding groups (≤ 1%). High defecation frequency was associated with looser stool consistency (*p* < 0.0001, lme).

Single-week findings of blood in the stool were reported in 9.3% (*n* = 98) and repeatedly in 5.2% (*n* = 55). At week 17, 2.6% (*n* = 25/946) reported blood. The proportion of infants with blood in stools was higher in the green stool group than in the total cohort (single-week reports in 17.3% (*n* = 26/150), *p* < 0.05, and repeated reports in 13.3% (*n* = 20/150), *p* < 0.005, respectively). Stool consistency (mean 6.1, SD 0.5) was comparable to the infants with no blood in stools. None of the infants with blood in the stool reported any specific diagnosis during the study period (e.g., inflammatory bowel disease, enteropathy, bleeding disorder).

### Defecation difficulty and crying

**Fig. 4:**
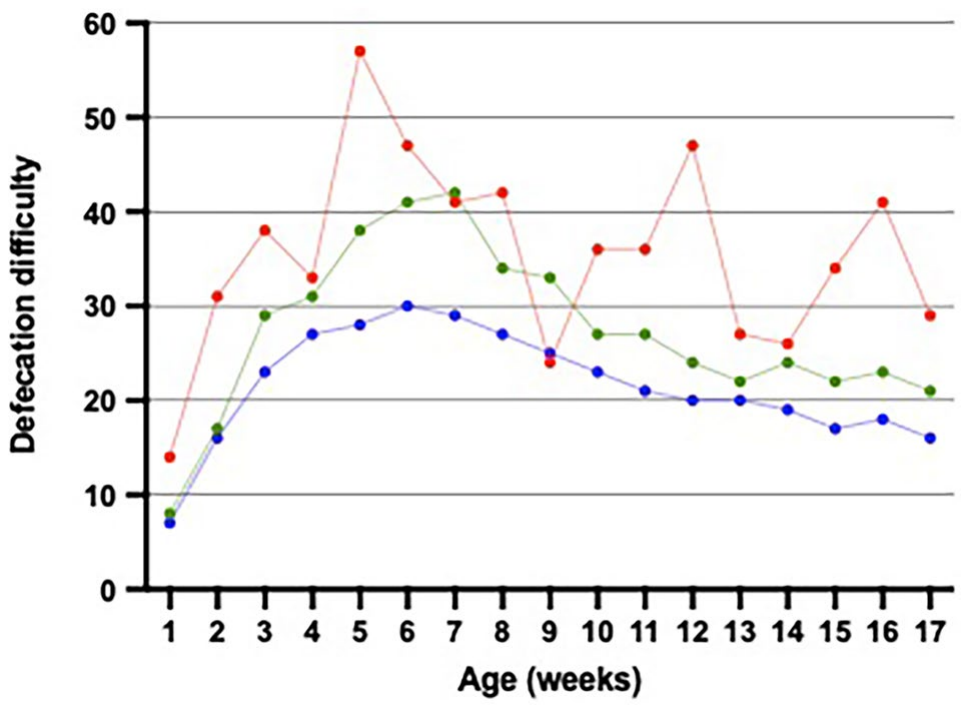
Defecation difficulty and the amount of daily crying in full-term infants during the first 17 weeks of life. The data of the 1052 infants were reported weekly online using visual analog scales (VAS) corresponding to numbers 1–100, with higher numbers indicating more symptoms. The dots represent median values. Group 1 with a daily cry of 0–2 h marked in blue, group 2 with a daily cry of 3–4 h marked in green, and group 3 with a daily cry of ≥ 5 h in red

Defecation was reported mostly as easy in the first 2 weeks and as most difficult at the age of 6 weeks (a median of 32, IQR 14–54). The group with the highest amount of crying at week 5 exhibited the highest defecation difficulty (Fig. 4 and eTable 2). Throughout the whole period, minor daily crying and looser stools were associated with easy defecation (*p* < 0.0001 and *p* = 0.007, lme). Moreover, formula-fed infants presented more symptomatic defecation than breastfed (a median of 27.9 compared to 22.2 during the entire 17-week period, in weeks 3 and 4 *p* < 0.05). However, when adjusting for defecation frequency, the effect of formula feeding became non-significant (*p* = 0.51, lme), as defecation difficulty was dependent on defecation frequency (*p* < 0.0001, lme).

The duration of daily crying was associated with age (nonlinearly, *p* < 0.0001), green stools (*p* < 0.0001), and feeding breastmilk from a bottle (*p* = 0.016), but not with defecation frequency (*p* = 0.37), stool consistency (*p* = 0.13), or formula feeding (*p* = 0.37).

## Discussion

We described the defecation patterns in a large prospective birth cohort including 1052 healthy term babies. Data were collected by interactive web-based questionnaires that parents filled up at weekly intervals throughout the 17 weeks of age providing unique real-life data. Most infants were breastfed, and defecation frequency was highest at the age of three weeks. The dominant color of the stool was most often yellow or light brown. However, nearly half of the infants had green stool dominating for at least 1 week during the first 17 weeks. Occasional blood was reported in almost 10%, a finding that was unexpected.

Previously, the defecation patterns of infants have been studied by observing a short period at a hospital, by 2-week follow-up at home, or in cross-sectional settings as comprehensively reviewed [2]. More recently, the defecation pattern and stool consistency in close to 500 infants were cross-sectionally recorded by using structured questionnaires covering the previous week [8], and in another study, including 600 term infants, parents reported defecation pattern and stool characteristics covering 3 days at 1, 2, and 3 months of age [11]. Our setting is unique as the families recorded the data every week. They answered questions about defecation frequency, defecation difficulty and color of stools, and perceived abdominal pain and crying of the infant. Defecation frequency was highest at the age of 3 weeks (median four times per day). Stools were most often yellow or light brown. Nearly black stools, most likely indicating meconium in the first days of life, were rare after the first week of life. When assessing defecation frequency and stool color concerning the feeding type, we verified the results of previous studies: breastfed infants presented more frequent defecation, and stools were more often yellow-colored than formula-fed infants [11, 18–21]. However, in our cohort, most infants were breastfed, and exclusive formula feeding was rare (2%) during the first 3 months. Our findings are well in line with the recent meta-analysis of defecation patterns in healthy children including young infants and children up to 4 years of age [22].

Interestingly, predominately green-colored stools were reported in almost every other child and reoccurring in approximately 15%. This is comparable to the previous reports showing that green-colored stools were a common finding and considered a normal phenomenon [11, 23]. In our cohort, blood was present in almost 10% of the infants at least during a single week, and up to 5% had repeated reports. We were not able to find any comparable reports in the literature. In the abovementioned Dutch study, infants with blood in stools were excluded [11]. Here, we found no health concerns in infants presenting with blood in stools, and stool consistency was like the other infants.

Previously, it was suggested that the green color in stools was associated with starting feeding with solids [18]. In our cohort, we did not observe such an association as most infants were not yet introduced to solids. It is a clinical interpretation that green stools reflect the increased transit time (perhaps deriving from a less fat content of the milk) and non-optimal feeding at that time point. By setting a criterion for 3 recurring weeks of green stools, we aimed to select the most representative group of infants to be compared to matched controls with no green stools. Infants with green stools seemed to present more abundant crying but had equal weight with control infants, and their health was good as routinely followed up at well-baby clinics. Reports of abdominal pain in the group of infants with repeated green stools were not different from the controls. Instead, the proportion of reports of blood among the stools was more frequent in this group than in the infants with no green stools although no reported difference in stool consistency.

Stool consistency was most often mushy or runny as measured with BSFS. Hard stools were rare (≤ 1%) in both the breastfed and formula-fed feeding groups. This is comparable to the previous studies [11, 23]. Stool form measured with the BSFS reflects mostly the whole gut transit time, whereas stool frequency does not show such an association [17]. However, feeding frequency may explain up to 5% of the variation in defecation frequency at the age of 1 month [11]. The average consistency of stool changes from soft toward hard with increasing age, the breastfed infants passing softer stools than formula-fed [18]. In some formulas, a combination of short-chain galacto-oligosaccharides and long-chain fructo-oligosaccharides has been introduced to mimic the composition of human milk oligosaccharides (HMOs). These prebiotics as well as probiotics (or synbiotics) that are present in some formulas (or may be taken as a supplement) may soften the stools, less often affecting the stool frequency [20, 24]. In our cohort, the type of formula was not considered, but some probiotic use (mostly intermittent) was reported in most of the infants by the age of 3 months as published [12, 13]. However, the observed stool frequency paralleled well with the previous publications [11].

Average daily crying time varied being most abundant at the age of 5 weeks. This is comparable to published knowledge stating that crying peaks at about 6 weeks are at its maximum during the first 3 months, and on average, about 2 h of crying per day is considered normal [25–27]. In our cohort, the infants with the highest crying at week 5 represented the group with the highest defecation difficulties and vice versa: infants with minor crying presented with low symptomatic defecation issues. Infants on formula feeding presented with more difficulties in defecation than breastfed infants. In all infants, defecation was easy during the first weeks, and the peak in reported difficulties occurred at week 6.

Data on bowel function and crying were based on parental reports on a visual scale. Notably, stool consistency was assessed with the Bristol scale which was designed to study stool consistency in adults. The evaluation was subjective; however, no validated objective measures to cover such a large study cohort were available. A recent study using photographs in the Brussels Infant and Toddler Stool Scale (BITSS) showed best-performing agreement with different observers in BSFS type 7 followed by type 6 [28], these being the most frequent scores of the parental evaluations in our cohort. When educating parents and medical professionals about the normal variation of infant bowel function and stool, it is important to include clear instructions on when to seek medical advice or referral to specialist care [2]. To detect acholic stools gray in color at an early age is essential for the prognosis of infants with biliary atresia [29, 30]. This may be challenging for the parents [29]. Therefore, the development of mobile applications with an ample selection of real-life images [31] has emerged to aid in the evaluation of the normality in infant stools.

### Strengths and limitations

This is the largest prospective cohort study describing the defecation pattern of term infants so far. Moreover, comprehensive data were collected, and weekly online and electronic questionnaires each week corresponded to the actual age of the infant. Questionnaires were mostly completed by the same person, i.e., the mother, thus limiting the individual variation in the repeated evaluations. In addition, 99% provided reports from routine visits at well-baby clinics. As a limitation, in such a large cohort, we did not ask for daily reports for 17 weeks, and instead, the participants reported weekly estimates. Notably, stool color is not constant, and the participants reported the most dominant color each week.

Despite some differences between the study group and the Finnish population [11], we believe that our findings on bowel function and crying habits may be considered representative of the healthy term infant population in a developed country.

## Conclusion

We utilized advanced electronic data collection each week rendering the available data comprehensive in a large cohort of term infants. Breastfed infants presented with frequent and yellow-colored stools as reported. Nearly half of the healthy term infants had green stool dominating for at least 1 week during the first 17 weeks. Occasional blood was reported in almost 10% with no difference in stool consistency during the first 17 weeks. Hard stools were rare. Our findings enlighten the spectrum of defecation patterns in healthy term infants during the first 17 weeks of life. A better understanding of bowel function helps healthcare professionals distinguish normal from abnormal when addressing defecation, the color of stools, and the type of feeding and may serve primary healthcare professionals when educating the families and caretakers of infants.

### Supplementary Information

Below is the link to the electronic supplementary material.

Supplementary file1 (DOCX 217 KB)

Supplementary file2 (DOCX 18.6 KB)

Supplementary file3 (DOCX 14.9 KB)

## Data Availability

Data is provided within the manuscript or supplementary information files. Non-identifiable data are available on a reasonable request.

## Abbreviations

BSFS: Bristol Stool Form Scale
FGID: Functional gastrointestinal disorder
HELMi cohort: Heath and Early Life Microbiota cohort
IQR: Interquartile range
VAS: Visual analog scale
