# Supplementary material for: Bowel function in a prospective cohort of 1052 healthy term infants up to 4 months of age
Source: Eur J Pediatr. 2024 May 31;183(8):3557–65. doi: 10.1007/s00431-024-05625-0 (PMC11263225; doi:10.1007/s00431-024-05625-0)
Supplement: Supplementary file 1 — Supplementary file1 (DOCX 217 KB) [file 431_2024_5625_MOESM1_ESM.docx]

**eFigure1** Defecation frequency in 1052 full-term infants concerning diet and stool color

Values represent the mean and 95% confidence interval.

**
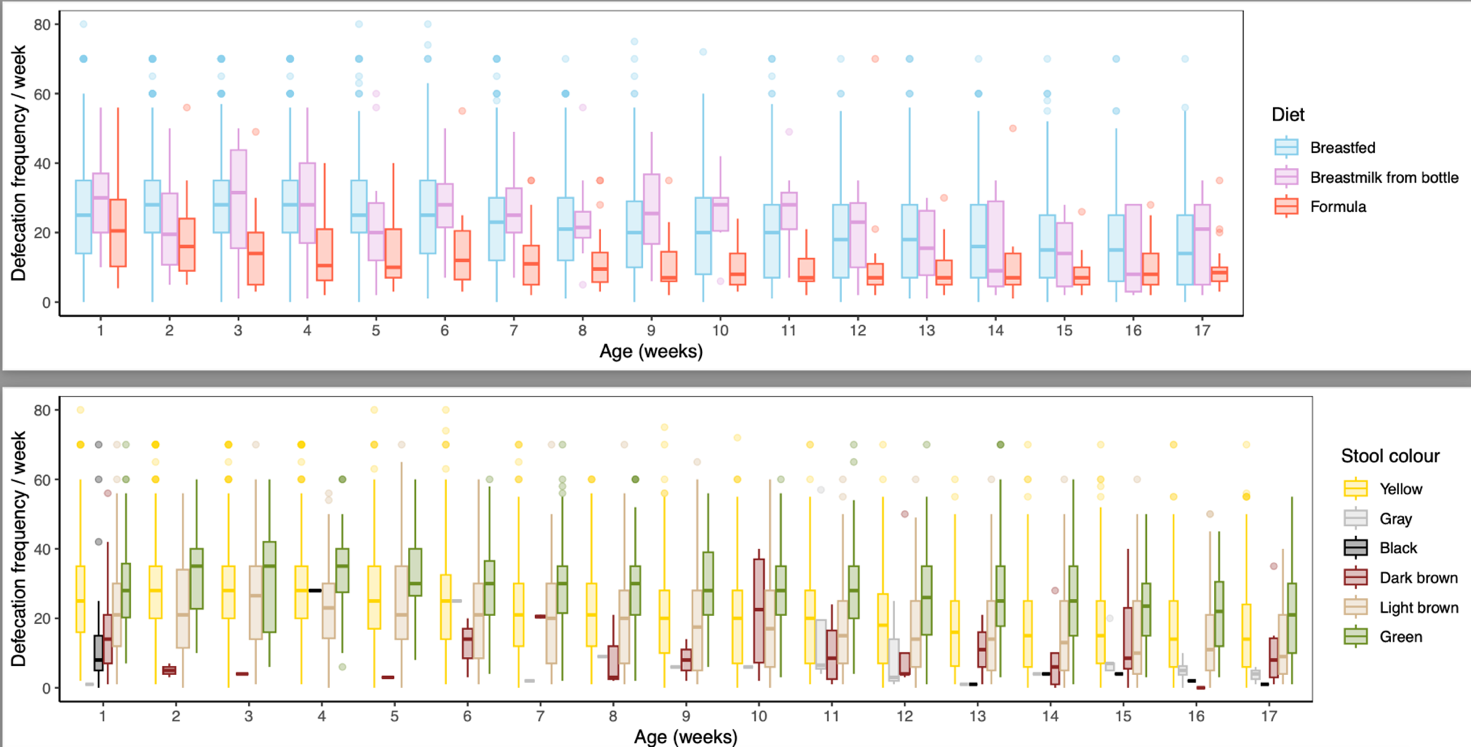
**
