# Supplementary material for: Bowel function in a prospective cohort of 1052 healthy term infants up to 4 months of age
Source: Eur J Pediatr. 2024 May 31;183(8):3557–65. doi: 10.1007/s00431-024-05625-0 (PMC11263225; doi:10.1007/s00431-024-05625-0)
Supplement: Supplementary file 2 — Supplementary file2 (DOCX 18.6 KB) [file 431_2024_5625_MOESM2_ESM.docx]

**eTable 1 Background data on the subgroups of infants and data points used in the analyses**

| **1.Matching according to predominant stool color** | **Total number of infants**  n= 444 |
| --- | --- |
| **Children with green-colored stool**, n  -Girl n (%)  -Breast milk n (%)  -Formula-fed n (%) | 150  75 (50)  145 (97)  5 (3) |
| **Children with yellow-colored stool**, n  -Girl n (%)  -Breast milk n (%)  -Formula-fed n (%) | 294  148 (50)  284 (97)  10 (3) |
| **2. Groups defined by daily crying** | **Total number of infants**  n=980 |
| **Group 1** with a daily cry of 0-2 hours; n  Boy, n (%)  Missing, n (%)  Breast milk n (%)  Cow-milk-derived formula, n (%) | 779  394 (50.6)  2 (0.2)  771 (99)  8 (1) |
| **Group 2**, with a daily cry of >2-4 hours; n  Girl, n (%)  Breast milk n (%)  Cow-milk derived formula, n (%)  Other formula, n (%) | 174  91 (52.3)  164 (94.3)  9 (5.2)  1 (0.5) |
| **Group 3**, with a daily cry of ≥ 5 hours; n  Boy, n (%)  Breast milk, n (%)  Cow-milk derived formula, n (%) | 27  14 (52)  26 (96.3)  1 (3.7) |
| **3. Data points used in linear mixed model analyses** | **Number of data points^a^** |
| Infant ID | 17079 |
| Duration of daily crying | 15237 |
| Defecation frequency | 15428 |
| Infant sex | 15519 |
| Defecation difficulty | 15429 |
| Stool color | 15428 |
| Feeding type | 16956 |
| **Birth mode** | 16299 |
| **4. Specific weekly questions** | Questionnaire each week for 0-17 weeks |
| Estimate the time during which the baby is crying per day |  |
| Intensity of crying | word anchors on a scale from 0 to 100 mm |
| The effect of soothing on a baby's crying | word anchors on a scale from 0 to 100 mm |
| Estimate of the number of times a week does the baby defecate |  |
| Effort necessary for the infant to defecate over the past week | word anchors on a scale from 0 to 100 mm |
| Classification of the appearance of stool over the past week | Bristol scale |
| Typical color of the stool over the past week | green/yellow/light brown/dark brown/clay gray/almost black |
| The child presented signs of GI symptoms and signs of stomach pains, flatulence, mucus or blood in stool, regurgitation, vomiting during the past week | true/false |
| The child presented signs of stomach pain during the past week | true/false |
| Intensity of the stomach pain during the past week | word anchors on a scale from 0 to 100 mm |
| The child presented signs of flatulence during the past week | true/false |
| Intensity of the flatulence pain during the past week | word anchors on a scale from 0 to 100 mm |
| The child presented mucus among the stool in the past week | true/false |
| The child presented blood among the stool in the past week | true/false |
| The child presented important regurgitation in the past week | true/false |
| Frequency of regurgitation over the past week | number per day |
| Amount of liquid regurgitated at each regurgitation events over the past week | word anchors on a scale from 0 to 100 mm |
| The child vomited in the past week | true/false |
| Frequency of vomiting over the past week | number per day |
| Proportion of the infant's diet composed of breastmilk in the past week | Group1: 0%; Group2: 1-30%; Group3: 31-70%; Group4: 71-95%; Group5: 96-100% |
| Infant's main source of nutrition in the past week | Group1: Breastmilk from a bottle; Group2: Breastmilk from the breast; Group3: Cow-milk based formula; Group4: Vegetal milk formula. |

^a^ The total number of data points used in the multivariate mixed-effects models with no missing data was 15207, which included data from 951 families.
