# Supplementary material for: Bowel function in a prospective cohort of 1052 healthy term infants up to 4 months of age
Source: Eur J Pediatr. 2024 May 31;183(8):3557–65. doi: 10.1007/s00431-024-05625-0 (PMC11263225; doi:10.1007/s00431-024-05625-0)
Supplement: Supplementary file 3 — Supplementary file3 (DOCX 14.9 KB) [file 431_2024_5625_MOESM3_ESM.docx]

**eTable 2**. Association of defecation difficulty and crying

|  | **Number of infants**  980 | **Defecation difficulty**  (on a scale of 0–100)  median (interquartile range) | *p*^a^ |
| --- | --- | --- | --- |
| **Group 1**  Daily crying time 0–2 hours/day | 779 | 28 (11–50) | < 0.0085  Group 1 vs. 3 |
| **Group 2**  Daily crying time >2–4 hours/day | 174 | 38 (22–60) | < 0.0001  Group 2 vs.1 |
| **Group 3**  Daily crying time ≥5 hours/day | 27 | 57 (21–72) | p=0.254  Group 3 vs. 2 |

^a^ the Kruskal-Wallis test
